# Serum metabolome and gut microbiome alterations are associated with low handgrip strength in older adults

**DOI:** 10.18632/aging.205501

**Published:** 2024-02-01

**Authors:** Yan Guo, Qin Wang, Yifan Lv, Fan Xia, Xin Chen, Yan Mao, Xiaodong Wang, Guoxian Ding, Jing Yu

**Affiliations:** 1 Department of Geriatrics, Division of Geriatric Endocrinology, The First Affiliated Hospital of Nanjing Medical University, Nanjing, P.R. China; 2 Department of Neurology, Yancheng City No. 1 People’s Hospital, Yancheng, P.R. China; 3 Department of Geriatrics, Drum Tower Hospital Affiliated to Nanjing University Medical School, Nanjing, P.R. China; 4 Department of Endocrinology, The First Affiliated Hospital of Nanjing Medical University, Nanjing, P.R. China

**Keywords:** metabolomics, metabolites, gut microbiota, omics integration, handgrip strength

## Abstract

Handgrip strength (HGS), which represents global muscle strength, is a powerful indicator of disability and mortality in older adults; it is also used for the diagnosis of possible- or probable- sarcopenia and physical frailty. This study aimed to explore the metabolic mechanisms and potential biomarkers associated with declining HGS among older adults. We recruited 15 age- and environment-matched inpatients (age, 77–90 years) with low or normal HGS. Liquid chromatography-mass spectrometry (LC-MS) and 16S ribosomal DNA (rDNA) gene sequencing were performed to analyze the metabolome of serum and stool samples and the gut microbiome composition of stool samples. Spearman’s correlation analysis was used to identify the potential serum and fecal metabolites associated with HGS. We assessed the levels of serum and fecal metabolites belonging to the class of cinnamic acids and derivatives and reported that the levels of carboxylic acids and their derivatives decreased in the low-HGS group. Serum levels of microbial metabolites, including cinnamoylglycine, 4-methoxycinnamic acid, and (e)-3,4,5-trimethoxycinnamic acid, were positively correlated with HGS. We found that gut microbial α-diversity was significantly higher in the low-HGS group, whereas higher β-diversity was observed in the normal group. The relative abundances of the genera *Parabacteroides* and *Intestinibacter* increased significantly in the low-HGS group and were negatively correlated with the serum levels of cinnamoylglycine. The identified metabolites whose levels were markedly altered, and intestinal flora associated with these metabolites suggest the potential metabolic underpinnings for HGS and provide a basis for the further identification of biomarkers of muscle strength decline in older adults.

## INTRODUCTION

Age-related skeletal muscle strength decline is an independent risk factor for high mortality in older adults [1]. Because handgrip strength (HGS) is easy to measure and can represent global muscle strength in older people [2], it is not only a powerful indicator of physical activity, nutritional status, and even disability [3], but can also be used for the diagnosis of sarcopenia [4, 5] and physical frailty [6]. The inverse association between HGS assessed at a single point in time and subsequent mortality (higher values are associated with lower mortality risk) has been established among older people [7, 8]. Moreover, the clinical value of HGS measured longitudinally to assess the risk of mortality among older people has also been validated [9]. Hence, understanding the biological underpinnings and identifying biomarkers for declining HGS that accompany aging is imperative.

Previous studies have found that inflammation-induced loss of muscle mass, elevated oxidative stress, mitochondrial dysfunction, and accumulation of advanced glycation end-products are involved in the mechanisms of, and serve as biomarkers for, muscle dysfunction-associated conditions, including muscle mass and strength loss. In fact, age-related decline in muscle strength does not parallel the decline in muscle mass [10]. Biomarkers reflecting muscle strength decline in the early stage have only been scarcely investigated, especially in older adults with normal or low HGS. In recent years, studies have shown that all the pathological bases of aging can cause metabolic reactions and that there is a “metabolic clock” that controls aging [11]. Thus, as products of metabolic reactions, metabolites play an important role in physiological and pathological aging [12, 13], making metabolomics an important tool for the non-invasive identification and quantification of biomarkers in biological matrices. However, studies using metabolomics to investigate the metabolites associated with HGS decline in older adults are limited.

The metabolic capacity of the human body is influenced by the gut microbiota and its interactions with host cells [14–16]. Gut microbiota dysbiosis, described as a profitless gut microbiota composition and diversity, has been proposed to occur during aging. Therefore, it has been proven that various age-related conditions, such as frailty and sarcopenia, are associated with the gut microbiome [17, 18]. The literature supports the possible presence of a “gut-muscle axis,” whereby gut microbial metabolism influences the functionality of muscle cells by producing mediators that drive the systemic effects of the gut microbiota [19, 20]. However, the potential relationship between the gut microbiome and metabolite profiles in older adults with low muscle strength is not well understood.

**Figure 1:**
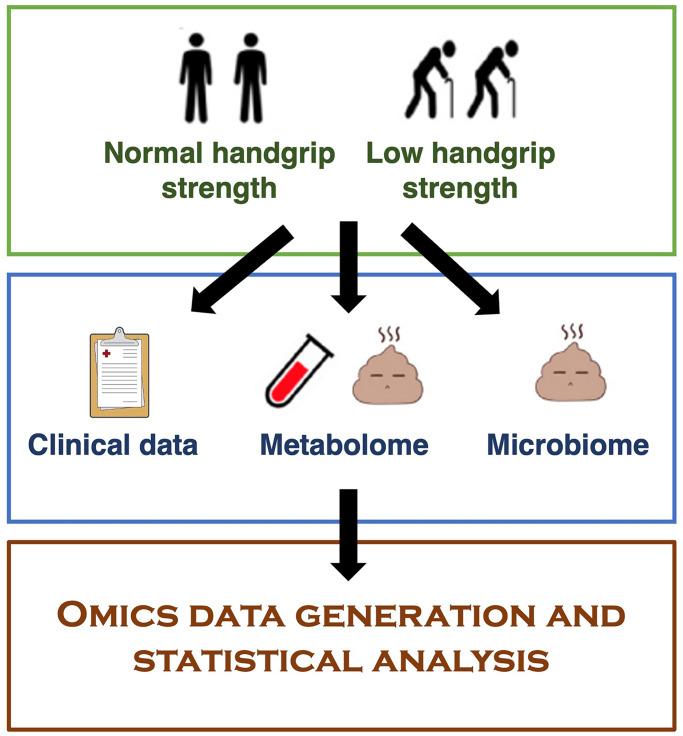
Overview of the study.

In this study, liquid chromatography-mass spectrometry (LC-MS) and 16S ribosomal DNA (rDNA) gene sequencing were performed to analyze the metabolome of serum and stool samples and the gut microbiome composition of stool samples from older adults with normal and low HGS. Correlation analysis was used to identify potential serum and fecal metabolites associated with the decline in HGS in older people. We further applied an integrated analysis of the microbiome and metabolome profiling to explore the correlations between altered gut microbiota and metabolites. The workflow is illustrated in Figure 1. The identified between-group differences for metabolites and their associated intestinal flora could indicate interactions between the host and gut microbiome in older adults with declining muscle strength.

## MATERIALS AND METHODS

### Clinical assessment

Individuals included in the study were recruited from the Division of Geriatric Endocrinology of the First Affiliated Hospital of Nanjing Medical University from 2019 to 2020 [21]. Patients with malignant tumors in the acute phase of the disease, those with severe cognitive impairment, and those taking antibiotics within the past month were excluded. Participants were given a form with questions concerning several lifestyle variables (smoking, alcohol consumption, comorbidities, and medications). Body mass index (BMI) was calculated based on height and weight measurements.

### Handgrip strength measurement

HGS of the dominant hand was measured using a portable hydraulic dynamometer (Jamar 5030J1, Jamar Technologies, Horsham, PA, USA). The participants adopted a seated upright position on a height-adjustable chair [22]. The test arm was positioned on a table to support the dynamometer’s weight. The participants were instructed and verbally encouraged to squeeze their handgrip as hard as possible. The best performance of the three trials was used in the analysis. Low HGS diagnostic cutoffs are <28.0 kg for men and <18.0 kg for women, according to the standard of the Asian Working Group for Sarcopenia (AWGS 2019) [4]. All measurements were performed by the same staff. Other methods for muscle mass measurement and physical performance, including the usual 4-m gait speed, 5-time chair stand test, and the Short Physical Performance Battery (SPPB), are specified in the Supplementary Materials and Methods.

### Blood and stool sample collection

Blood samples were collected from all participants by nurses on the second day of overnight fasting. Samples were centrifuged at 3,000 rpm for 10 min at 4°C to obtain the serum, and then aliquoted and stored at −80°C until further analyses. Stool samples were collected within three days of admission using a Commode specimen collection system (Thermo Fisher Scientific, Waltham, MA, USA), aliquoted, frozen immediately in liquid nitrogen, and then stored at −80°C before further processing.

### Untargeted metabolomics analysis of serum and stool samples

All the samples were thawed on ice before extraction using a solvent with a vortex or grinding step before centrifugation. After centrifugation, the reconstituted solution was added to the supernatant with a 60-s vortexing step before centrifugation for reconstitution. The supernatants were collected for metabolomic profiling using LC-MS analysis. A quality control (QC) sample was prepared by mixing equal volumes of the supernatant of each sample to assess the analytical variability. Untargeted metabolomics LC-MS analysis was performed, and data were collected in both the positive- and negative-ion modes to improve the coverage of metabolites. Refer to the Supplementary Materials and Methods for further details.

Raw LC-MS data were extracted and processed using multivariate statistical analysis (Partial Least Squares Method-Discriminant Analysis, PLS-DA) to establish a relationship model between the metabolite and sample groups. Univariate methods (Wilcoxon test and two-tailed Student’s *t*-test) were used to detect the metabolites whose levels were significantly altered; then, correction was performed by calculating the false discovery rate (FDR) to ensure that the metabolite peaks were reproducibly detected. Metabolites responsible for the difference in the metabolic profile scan between the groups were obtained based on a variable importance in the projection (VIP) threshold of 1 from the 7-fold cross-validated PLS-DA model. By combining the univariate and multivariate statistical analyses, metabolites whose levels were significantly altered between groups were acquired under the following conditions: *p*-value < 0.05, *q*-value < 0.05, fold change < 0.8 or > 1.2, and VIP > 1.

The online HMDB database (http://www.hmdb.ca) was used to annotate metabolites by matching the exact molecular mass data (m/z) of the samples with those from the database [23]. The KEGG database (https://www.genome.jp/kegg/) was used to understand the functional characteristics of the differential metabolites and to determine the main biochemical metabolic pathways and signal transduction associated with the metabolites. A hypergeometric test was used to identify the significantly enriched pathway entries. Metabolic pathways with a *p*-value < 0.05 were deemed to be significantly enriched by differential metabolites.

### 16S rDNA microbiome analysis of stool samples

DNA was extracted from all fecal samples and the 16S rDNA was PCR-amplified and sequenced on the MiSeq system (Illumina, San Diego, CA, USA). The sequence reads with a similarity greater than 97% were identified for further analysis. Operational taxonomic unit (OTU) representative sequences were taxonomically classified using the Ribosomal Database Project (RDP) Classifier, and community composition was analyzed for each taxonomic rank: domain, kingdom, phylum, class, order, family, genus, and species. The complete protocol can be found in the Supplementary Materials and Methods.

### Spearman’s multiomics correlation analysis

Spearman correlation analysis based on the R package (v3.4.1) was used to analyze the correlation between the metabolites and microbiota. Spearman’s correlation coefficients were also computed for the relationships between the HGS values and individual metabolomic features. Differences were considered significant when the *p*-value was < 0.05 and |r| was > 0.5. If r < 0, there was a negative correlation; otherwise, there was a positive one.

### Data availability

Raw LC-MS data are available in the MetaboLights repository (accession no. MTBLS4367 and MTBLS4372). The sequencing data of the 16S rDNA were deposited in the BioProject database (accession no. PRJNA787524).

## RESULTS

### Clinical data of study participants

Eight inpatients with low HGS and seven age- and environment-matched inpatients with normal HGS (age, 77–90 years) were recruited. HGS is an important component of sarcopenia diagnosis and a representative indicator of muscle strength. Thus, we also collected information regarding other components of sarcopenia, including appendicular skeletal muscle (ASM), 4-m gait speed, 5-time chair-stand test, and SPPB (Table 1). The clinical characteristics of the study participants are shown in Table 2. There were no statistically significant differences between the two groups with regard to age, BMI, health behaviors, ASM, 4-m gait speed, or SPPB. The total number of comorbidities and medications, especially, diabetes and antidiabetic medications, was higher in the low-HGS group than in the normal group. Similar to the HGS, a 5-time chair stand test was another evaluation method used to determine “Possible sarcopenia”. The time for the test increased in the low-HGS group.

**Table 1:** Sarcopenia-associated parameters in accordance with handgrip strength in 15 participants.

**Normal HGS**						
**Gender**	**Age (years)**	**Muscle strength**	**Muscle mass**	**Physical performance**		
		**HGS (kg)**	**RASM (kg/m^2^)**	**4-m gait speed (m/s)**	**5-time chair stand test (s)**	**SPPB**
M^†^	82	29.80	5.80	0.99	17.01	8
M^†^	81	29.90	6.06	1.20	15.40	10
M	79	29.93	7.07	1.26	11.18	12
M	83	31.10	6.41	1.72	9.39	12
M^†^	87	34.30	5.56	0.80	-	5
F^†^	82	20.00	5.35	1.29	11.55	9
F^†^	77	20.10	4.75	1.15	-	8
Avg ± std	81.57 ± 3.15	27.88 ± 5.57	5.86 ± 0.75	1.20 ± 0.28	12.91 ± 3.17	9 ± 2
**Low HGS (Male: < 28 kg, Female: < 18 kg)**						
**Gender**	**Age (years)**	**Muscle strength**	**Muscle mass**	**Physical performance**		
		**HGS (kg)**	**RASM (kg/m^2^)**	**4-m gait speed (m/s)**	**5-time chair stand test (s)**	**SPPB**
M^‡^	81	27.10	4.66	1.04	18.30	9
M^‡^	90	18.80	5.45	-	-	0
M^‡^	84	15.00	5.95	-	-	0
M^‡^	80	26.67	5.38	0.72	18.97	6
M^‡^	90	23.37	5.19	1.16	15.34	10
M^‡^	85	24.40	5.98	0.98	24.50	9
F^*^	80	15.90	5.98	1.26	12.07	11
F^*^	87	10.37	5.82	0.57	23.17	4
Avg ± std	84.63 ± 4.14	20.20 ± 6.11	5.55 ± 0.47	0.95 ± 0.26	18.73 ± 4.67	6 ± 4
*P*-value	0.1364	0.0252	0.3559	0.1364	0.0427	0.1326

Abbreviations: M: male; F: female; HGS: handgrip strength; RASM: relative appendicular skeletal muscle mass; SPPB: Short Physical Performance Battery; -: Participants cannot complete the test. ^*^: Possible sarcopenia, ^†^: Sarcopenia, ^‡^: Severe sarcopenia (according to the diagnosis criteria of Asian Working Group for Sarcopenia, 2019).

**Table 2:** Clinical characteristics of the study participants.

	**Handgrip strength**		***P*-value**
	**Normal (*n* = 7)**	**Low (*n* = 8)**	
**Age, years**	81.57 ± 3.15	84.63 ± 4.14	0.1364
**Sex (male/female)**	5/2	6/2	N/A
**BMI, kg/m^2^**	24.00 ± 1.61	23.26 ± 2.43	0.5062
**Health behavior (*n* (%))**			
Smoking	2 (29%)	3 (38%)	0.7144
Alcohol drinking	1 (14%)	2 (25%)	0.6048
**Total number of comorbidities (*n* (%))**	0.96 ± 0.11	2.75 ± 0.37	**0.0024**
Hypertension	2 (28.57%)	5 (62.50%)	0.1888
Diabetes mellitus	0 (0%)	5 (62.50%)	**0.0104**
Stroke	0 (0%)	3 (37.50%)	0.0701
Coronary artery disease	0 (0%)	1 (12.50%)	0.3329
**Total number of medications (*n* (%))**	1.43 ± 0.53	5.25 ± 0.84	**0.0026**
Psycho-drugs	0 (0%)	0 (0%)	N/A
Antidiabetic agent	0 (0%)	5 (62.50%)	**0.0104**
Statin	3 (42.86%)	1 (12.50%)	0.1847
β-blocker	0 (0%)	1 (12.50%)	0.3329
Antiplatelet	2 (28.57%)	1 (12.50%)	0.4376
ACEi and/or ARB	1 (14.29%)	3 (37.50%)	0.3104
β2 sympathomimetic inhaler	0 (0%)	0 (0%)	N/A

### Serum metabolomics analysis

**Figure 2:**
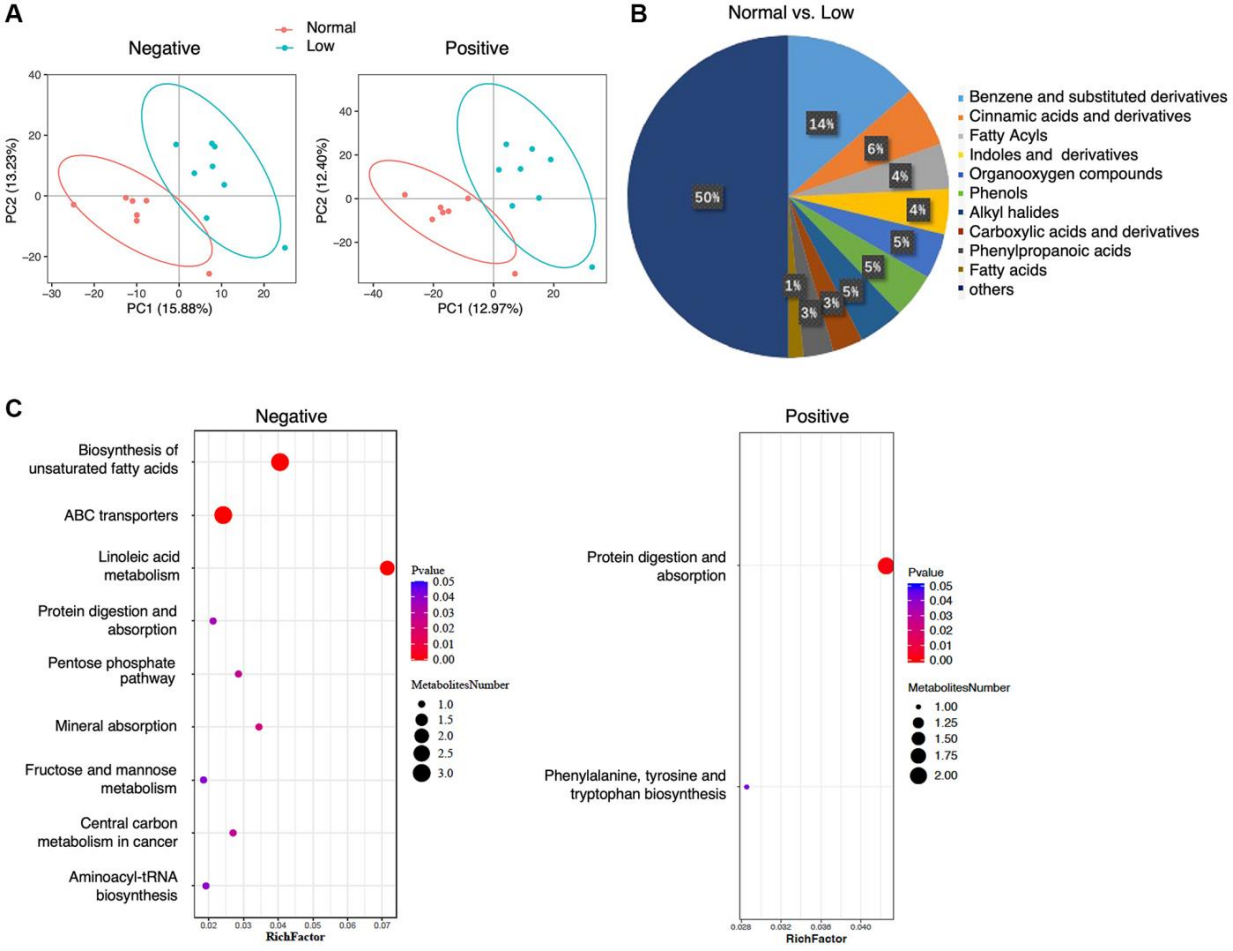
**Serum metabolomics analysis.** (**A**) PLS-DA analysis of the grouped discrimination by the first two principal components (PCs) in negative and positive ion modes. (**B**) Pie graph of the class composition according to the number of altered metabolites. (**C**) Bubble chart of the pathway enrichment analysis of differential metabolites analyzed in the negative- and positive-ion modes. RichFactor was the number of differential metabolites divided by the identified metabolites annotated to the pathway.

Metabolomics analysis yielded 386 metabolites in the negative-ion mode and 861 metabolites in the positive-ion mode. Supervised PLS-DA revealed that the metabolomic profiles differed between the normal and low HGS groups (Figure 2A). In total, the abundance of 67 annotated metabolites was found to be significantly different between the two groups, with 25 metabolites having higher concentrations and 42 metabolites having lower concentrations in the serum of older adults with low HGS (Supplementary Table 1). Class information was available for only 47 of these 67 metabolites, according to the Human Metabolome Database (HMDB version 5.0). Analysis of these metabolites showed that compared with the normal group, the shifts in the low-HGS group mainly included benzene and their (substituted) derivatives, cinnamic acids and their derivatives, and fatty acids (Figure 2B). In particular, the levels of metabolites belonging to the class of cinnamic acids and their derivatives were lower in the serum of older adults with low HGS, while the levels of metabolites belonging to the class of fatty acids were higher in the normal group (Supplementary Table 1). KEGG metabolic pathway analysis revealed a significant enrichment of 10 pathways, including linoleic acid metabolism, biosynthesis of unsaturated fatty acids, and ABC transporters (Figure 2C, Supplementary Table 2). The results revealed alterations in serum metabolic characteristics in subjects with decreased HGS.

**Figure 3:**
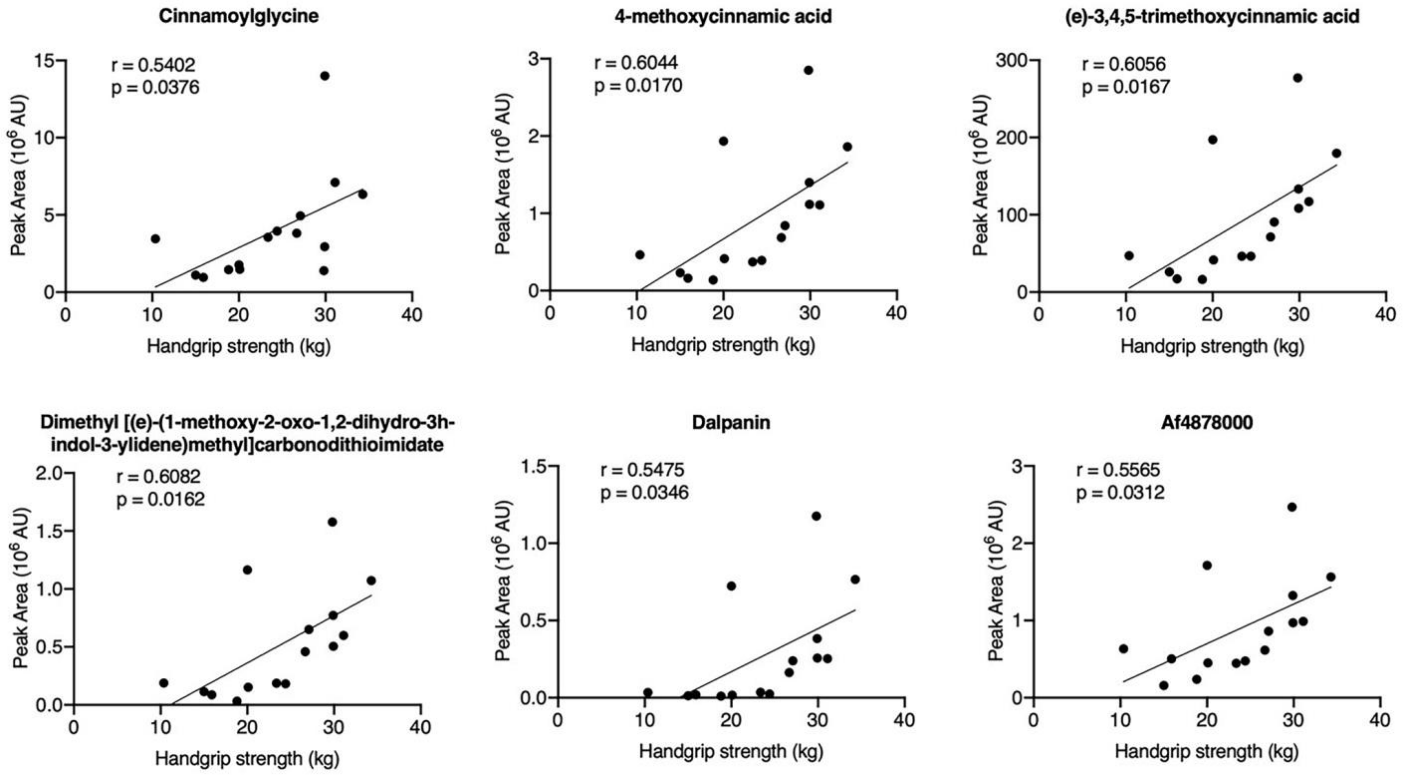
Correlation analysis of the HGS values and differential metabolites in serum samples.

To evaluate the relationship between the HGS value and the 67 annotated metabolites, Spearman’s correlation analysis was performed. Six metabolites were positively correlated with the HGS values: cinnamoylglycine, 4-methoxycinnamic acid, (e)-3,4,5-trimethoxycinnamic acid, dimethyl ((e)-(1-methoxy-2-oxo-1,2-dihydro-3h-indol-3-ylidene)methyl)carbonodithioimidate, dalpanin, and Af4878000 (Figure 3). Among these metabolites, the appearance of cinnamoylglycine, 4-methoxycinnamic acid, and (e)-3,4,5-trimethoxycinnamic acid suggested that cinnamic acid metabolism may play a role in the decline of HGS in older adults.

### Fecal metabolomics analysis

**Figure 4:**
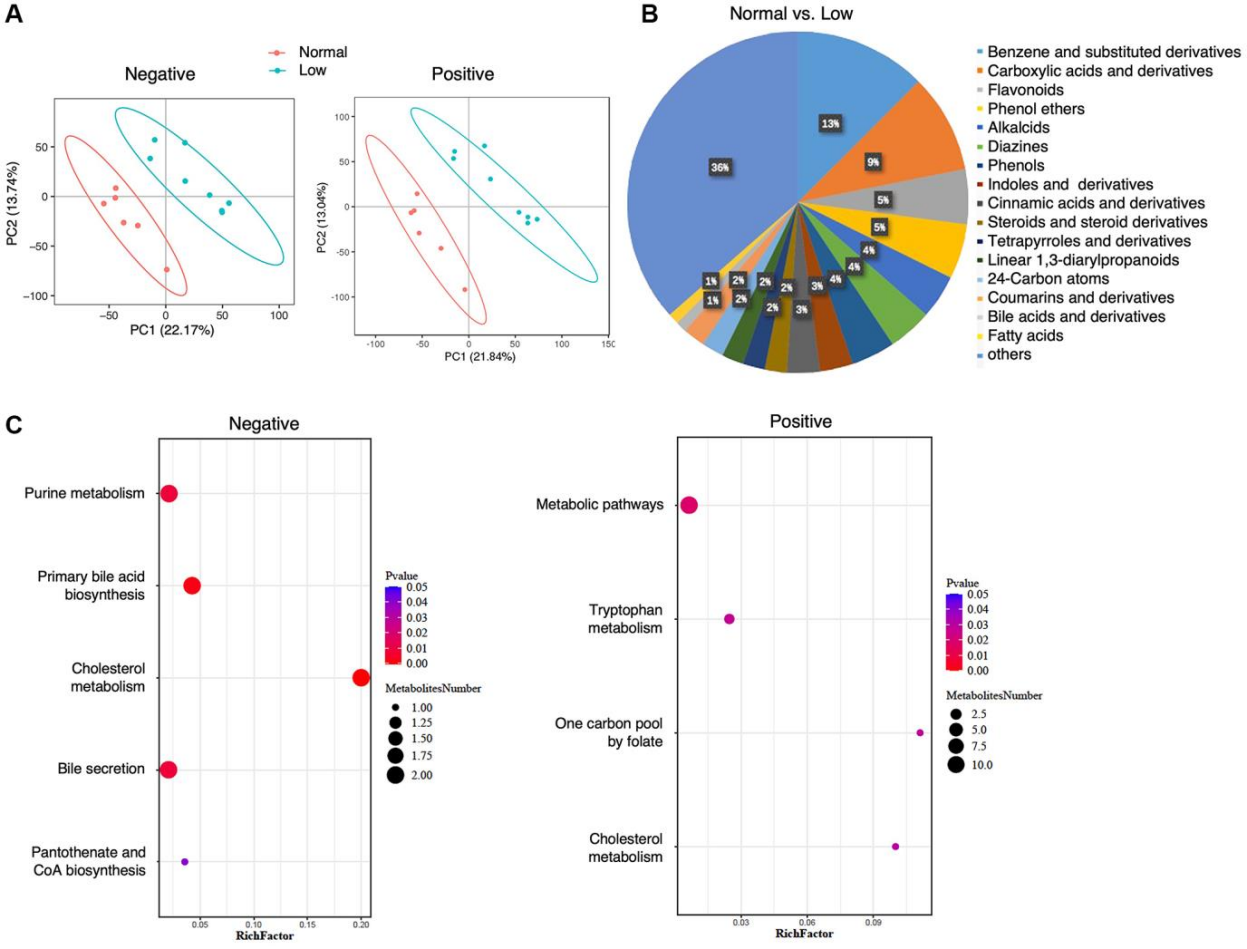
**Fecal metabolomics analysis.** (**A**) PLS-DA analysis of the grouped discrimination by the first two principal components (PCs) in the negative- and positive-ion modes. (**B**) Pie graph of the class composition according to the number of altered metabolites. (**C**) Bubble chart of pathway enrichment analysis of differential metabolites analyzed in the negative- and positive-ion modes. RichFactor was the number of differential metabolites divided by the identified metabolites annotated to the pathway.

In total, 1268 metabolites were identified in the negative-ion mode and 2255 metabolites were identified in the positive-ion mode. PLS-DA showed a clear separation trend between the normal and low HGS groups (Figure 4A). All fecal metabolites with annotations that were higher (*n* = 78) or lower (*n* = 99) in the low-HGS group are shown in Supplementary Table 3. Analysis of 96 metabolites for which class information was available revealed that the levels of fecal metabolites were altered in the low-HGS group; these metabolites mainly included benzene and its (substituted) derivatives, carboxylic acids and their derivatives, flavonoids, and phenol ethers (Figure 4B). Moreover, the relative concentration of metabolites belonging to the class of cinnamic acids and their derivatives also decreased in the feces of older subjects with low HGS (Supplementary Table 3). KEGG metabolic pathway mapping showed that the metabolites were significantly enriched in seven pathways, including cholesterol metabolism, primary bile acid biosynthesis, and bile secretion (Figure 4C, Supplementary Table 4).

Similarly, to determine the differences that could be associated with HGS, we examined the correlations between the relative concentrations of the annotated metabolites and HGS values. Thirteen metabolites were found to be associated with changes in HGS values, of which four were positively correlated with HGS and nine were negatively correlated with HGS (Supplementary Figure 1). Most of the metabolites were drugs and their metabolic derivatives, such as sitagliptin, pioglitazone, emedastine, and venlafaxine. Gamma-glu-gln and histidylphenylalanine, which were included in the class of carboxylic acids and their derivatives, showed a positive correlation with HGS. Indole-2-carboxylic acid was positively correlated with HGS. Importantly, one metabolite from the class of cinnamic acids and their derivatives, (2e)-5-hydroxyferulic acid, was also positively correlated with HGS. These results suggest that the fecal metabolite profiles between the normal and low-HGS groups differed.

### Microbiome analysis

**Figure 5:**
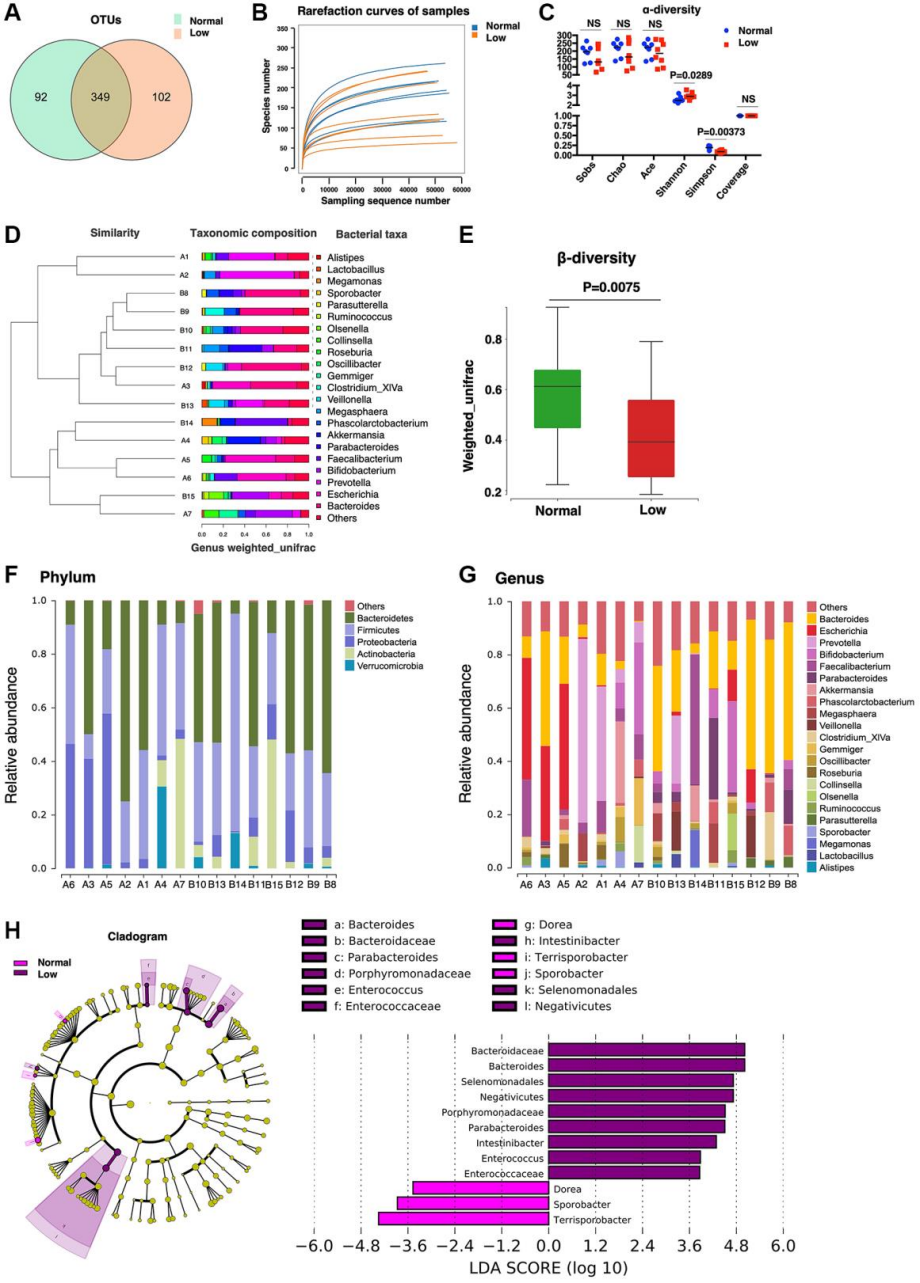
**Microbiome analysis.** (**A**) The Core-Pan graph of OTU distribution between the two groups. (**B**) The rarefaction curve of random sequences per sample and their corresponding number of observed species. (**C**) Species diversity differences estimated on the basis of the observed Sobs, Chao, Ace, Shannon, Simpson, and Coverage indices. (**D**) UPGMA cluster analysis of 15 samples at the genus level. A1–A7 represent the normal handgrip strength group; B8–B15 represents the low handgrip strength group. (**E**) β-diversity box-plot constructed on the basis of Weighted UniFrac analysis between the two groups. (**F**) The percentages of gut microbiota diversity at the phylum level. (**G**) The percentages of gut microbiota diversity at the genus level. (**H**) Linear discriminant analysis (LDA) integrated with effect size (LEfSe). Left: the phylogenetic distribution of microbiota in the Cladogram. Right: the differences in the abundance of microbiota.

Previous studies demonstrated a surprisingly notable effect of the gut microbiome on mammalian blood metabolites [24] and approximately 58% of metabolites detected in human body originate from microbiota [25]. To identify the changes in the gut microbiota of older adults with low HGS, we performed 16S rDNA amplicon sequencing of fecal samples from 15 participants. In total, 543 OTUs were detected, and 349 OTUs overlapped between the normal and low-HGS groups (Figure 5A). The number of unique OTUs in the low-HGS group was slightly higher than that in the normal group, indicating a more abundant microbiota in individuals with low HGS. According to the species associated OTUs and sequence number, rarefaction curves of the samples were calculated, and a flat trend indicated that the sampling size was reasonable (Figure 5B). Gut microbial α-diversity was lower in the low-HGS group than in the normal group, as calculated using the Simpson indices (Figure 5C). For β-diversity, UPGMA cluster analysis based on weighted UniFrac analysis was performed, and the phylogenetic distance between the samples was calculated (Figure 5D). We observed a higher β-diversity in the gut microbiota of older adults with normal HGS, indicating a more heterogeneous community structure among participants with normal HGS than among those with low HGS (Figure 5E).

Next, the gut microbial composition abundances in the stool samples were analyzed at the phylum and genus levels (Figure 5F, 5G). At the phylum level, Bacteroidetes and Firmicutes were the predominant phyla. There were no significant differences in microbiome composition abundance at the phylum level between the normal and low-HGS groups. At the genus level, the relative abundance of the genera *Parabacteroides* and *Intestinibacter* increased in the low-HGS group (Table 3). Furthermore, we used linear discriminant analysis effect size (LEfSe) to generate a cladogram to identify specific bacteria associated with decreased HGS. The results showed that members from *Bacteroidaceae*, *Bacteroides*, *Selenomonadales*, Negativicutes, *Porphyromonadaceae*, *Parabacteroides*, *Intestinibacter*, *Enterococcus*, and *Enterococcaceae* were the most abundant microbiota in the low-HGS group (all | Linear discriminant analysis (LDA) scores (log10) | > 3.6). However, participants with normal HGS were mainly characterized by higher abundances of *Sporobacter* and *Terrisporobacter* (all | LDA scores (log10) | > 3.6) (Figure 5H). The data revealed pronounced differences in microbiota between the two groups.

**Table 3:** Relative abundance of fecal microbiota at the genus level.

**Bacteria species**	**Normal HGS**	**Low HGS**	***P*-value**	**FDR**
	**Relative abundance (%)**			
*Parabacteroides*	0.400073	6.889853	0.006536	0.466388
*Intestinibacter*	0.004498	0.027854	0.008969	0.466388

### Correlations between gut microbiota and serum/fecal metabolites

**Figure 6:**
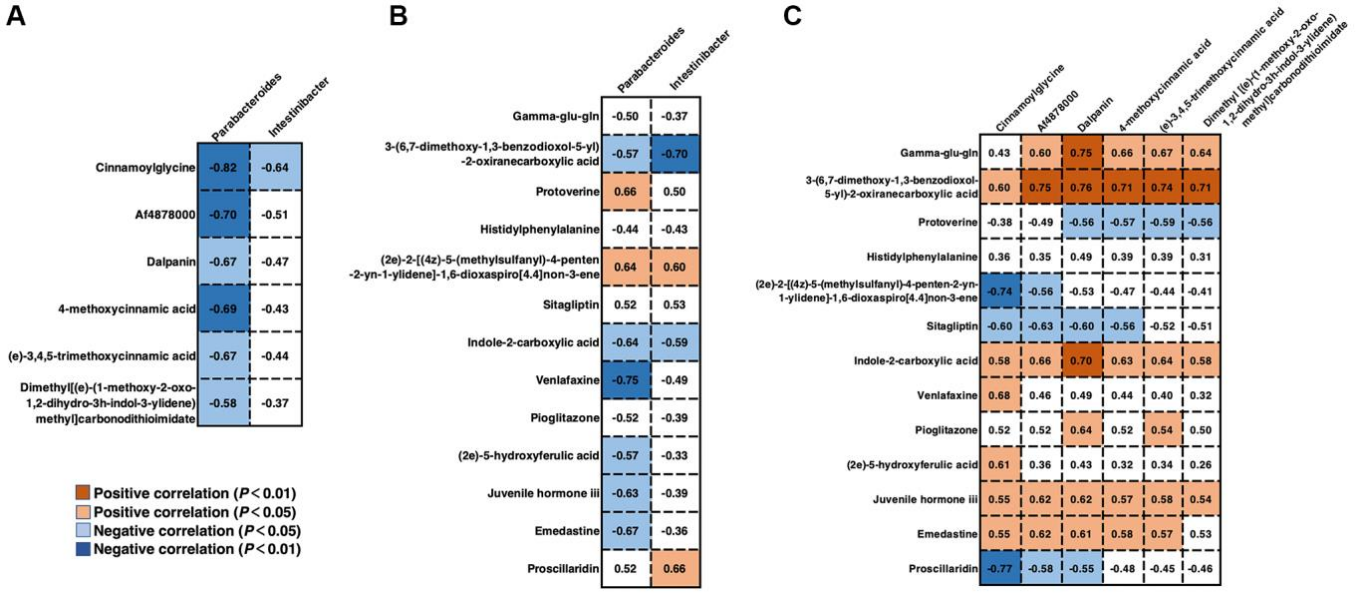
**Correlations between gut microbiota and serum/fecal metabolites.** (**A**) Spearman’s correlation coefficient between the genera *Parabacteroides* and *Intestinibacter* and serum metabolites. (**B**) Spearman’s correlation coefficient between the genera *Parabacteroides* and *Intestinibacter* and fecal metabolites. (**C**) Spearman’s correlation coefficient between the serum and fecal metabolites. Differences were considered significant when the *p*-value was < 0.05 and |r| was > 0.5. If r < 0, there was a negative correlation.

To explore the potential dependencies between microbiome composition, host metabolism, and the metabolome, we examined the correlations between the two datasets. Spearman’s correlation coefficient was computed between the relative abundance of HGS-associated species at the genus level and the different serum and fecal metabolites. Enrichment of members from the genus *Parabacteroides* in stool samples from older adults with low HGS was negatively correlated with the six serum metabolites whose levels increased with the HGS value (Figure 6A). The correlation coefficient between cinnamoylglycine and *Parabacteroides* was the most significant (|r| = 0.82). Moreover, *Intestinibacter*, whose abundance also increased in older adults with low HGS, correlated negatively with cinnamoylglycine. For fecal metabolites associated with low HGS, the abundance of *Parabacteroides* was also negatively correlated with (2e)-5-hydroxyferulic acid, a cinnamic acid derivative (Figure 6B). The abundances of the genera *Parabacteroides* and *Intestinibacter* were negatively correlated with indole-2-carboxylic acid. Correlations between serum and fecal metabolites altered by HGS were also analyzed. Six serum metabolites showed positive correlations with HGS and were positively correlated with fecal indole-2-carboxylic acid and gamma-glu-gln levels. Serum cinnamoylglycine was positively correlated with fecal (2e)-5-hydroxyferulic acid, which also belongs to the class cinnamic acids and their derivatives (Figure 6C). These results indicated that alterations in the levels of serum metabolites and their related fecal metabolites in the low-HGS group may be associated with the gut microbiota.

## DISCUSSION

Declining muscle strength with aging is almost inevitable; however, the metabolic mechanisms underlying this phenomenon remain poorly understood. Using an LC-MS-based metabolomics approach, we investigated the serum and fecal metabolites whose levels were altered in older adults with normal and low HGS. In addition to the metabolites belonging to the class of benzene and its (substituted) derivatives, the shifts in the low-HGS group mainly included cinnamic acids and derivatives. 4-methoxycinnamic acid, (e)-3,4,5-trimethoxycinnamic acid, sinapinic acid, and (2e)-3-(3,4-dimethoxyphenyl) acrylic acid were the four metabolites belonging to the class of cinnamic acids and their derivatives, and their relative concentrations decreased in the serum of older adults with low HGS. In particular, 4-methoxycinnamic acid and (e)-3,4,5-trimethoxycinnamic acid levels were positively correlated with HGS. Fecal metabolites of this class also decreased in the low-HGS group. Cinnamic acids and their derivatives, both synthetic and derived from natural sources, have been reported to exert a wide range of biological activities, including anticancer, hepatoprotective, neuroprotective, cardioprotective, and antidiabetic effects, and potent functions in muscle cell proliferation, differentiation, and development [26–30]. Sinapinic acid is one of the most common hydroxycinnamic acids; it is widespread in the plant kingdom [31]. It has also been reported to be a major active component of traditional Chinese remedies [32]. The antioxidant and anti-inflammatory activities of sinapinic acid have been reported to be highly significant. Sinapinic acid scavenges native peroxynitrite (ONOO^−^) and is more efficient than its alkyl esters [33, 34]. Lee et al. [35] showed that sinapinic acid modulates inflammation by suppressing NOD-like receptor pyrin domain-containing 3 (NLRP3) inflammasome activation. In addition, supplementation with sinapinic acid affected the intestinal microbiome by improving the proportion of the butyrate acid producers *Blautia* and *Dorea* and inhibiting the growth of bacterial species associated with diseases and inflammation, such as *Bacteroides* [36]. Although further studies on other cinnamic acids and their derivatives are needed, the above findings, including those of our study, fully suggest the potential role of this class of metabolites in aging and related diseases.

Cinnamoylglycine, another metabolite involved in the metabolism of cinnamic acid, is a glycine conjugate of cinnamic acid; it belongs to the class of carboxylic acids and their derivatives. In the present study, the relative concentration of cinnamoylglycine decreased with the decrease in the HGS. We also found that the levels of hippurate, a glycine conjugate of benzoic acid, decreased in the serum of older adults with declining HGS. These two metabolites are derived from catabolism of dietary polyphenols found in plant-based foods, which is performed by the intestinal microflora [24, 37]. Hippuric acid in plasma has been recognized as a plausible hallmark of frailty and geriatric syndromes [38]. Lustgarten et al. found that cinnamoylglycine, which is related to gut bacterial metabolism, was negatively associated with muscle quality [39] and physical function in older adults [40]. Cinnamoylglycine also predicted a higher gut microbiome diversity and was linked to a lower incidence of type 2 diabetes [41]. Although the current findings regarding the role of cinnamoylglycine in muscle strength and body function are inconsistent, cinnamoylglycine has the potential to serve as a biomarker of age-related changes in muscle strength; this warrants further studies.

A few integrated studies regarding the relationship between serum, fecal metabolomics, and gut microbiome and muscle dysfunction have been published [42, 43]. The microbial species and their associated metabolites involved in HGS-associated alterations were identified in the present study. We found that the abundance of two genera, *Parabacteroides* and *Intestinibacter*, which belong to the phyla *Bacteroidetes* and *Firmicutes*, respectively, increased in the low-HGS group. The two gut commensal bacteria have been reported to produce short-chain fatty acids [44, 45]. *Parabacteroides* produces acetate to reduce neutrophil infiltration [45] and can be affected by prebiotic supplementation in frail older subjects [46]. The metabolic benefits of *Parabacteroides distasonis* in decreasing weight gain, hyperglycemia, and hepatic steatosis have been previously studied [47]. A higher abundance of *Intestinibacter*, which can produce butyrate, is associated with the reduced incidence of type 2 diabetes [44] and its abundance decreases immediately after metformin treatment [48, 49]. A recent study on coronavirus disease 2019 (COVID-19) found that *Intestinibacter bartlettii* was positively correlated with anorexia and fatigue in survivors of COVID-19 after discharge from the hospital [50]. We found that the members of *Parabacteroides*, which were enriched in stool samples of low-HGS older adults, were negatively related to six serum metabolites that were associated with HGS, including cinnamoylglycine, a cinnamic acid derivative. The abundance of *Parabacteroides* was also negatively correlated with fecal (2e)-5-hydroxyferulic acid, which is a cinnamic acid derivative. The abundances of members from *Parabacteroides* and *Intestinibacter* were negatively correlated with the fecal metabolite indole-2-carboxylic acid. The discovery of these microbial metabolites and their correlated microbiota in older adults with low HGS indicates that microbial metabolites, along with other traditional markers, can be used as potential biomarkers for muscle strength decline.

This study has limitations including small sample size. Although environmental-matched controls were recruited and participated, an important limitation was the lack of dietary control among participants. Dietary patterns consistently correlate with groups of bacteria with shared functional roles in both health and disease [51]. The influence of dietary protein on the gut microbiome and its impact on sarcopenia also has been explored [52]. We will expand the sample size and consider various influencing factors, including diet, in future studies.

In summary, this work demonstrates that the levels of serum and fecal metabolites, especially, gut microbiota-related metabolites, are notably altered in older people with low HGS. The findings of the current study suggest potential metabolic underpinnings for HGS and provide a basis for the further identification of biomarkers of muscle strength decline in older adults.

## Supplementary Materials

Supplementary Materials and Methods

Supplementary Figure 1

Supplementary Tables 1 and 3

Supplementary Tables 2 and 4
