# Supplementary material for: Serum metabolome and gut microbiome alterations are associated with low handgrip strength in older adults
Source: Aging (Albany NY). 2024 Feb 1;16(3):2638–56. doi: 10.18632/aging.205501 (PMC10911350; doi:10.18632/aging.205501)
Supplement: Supplementary Materials and Methods [file aging-16-205501-s001.pdf]

Supplementary Materials and Methods

Skeletal muscle mass measurement

Limb fat-free mass (the sum of lean mass in the arms and legs) obtained from whole-body dual-energy X-ray absorptiometry (DXA; Hologic Inc., Bedford, MA, USA) was used to quantify for appendicular skeletal muscle (ASM) mass. Height-adjusted relative appendicular skeletal muscle mass (RASM) was calculated (ASM/ height^2, kg/m^2).

Physical performance

4-m gait speed

Gait speed was evaluated using a 4-meter walk, which served as an indicator of physical performance. The participants walked straight for 4 m at their usual pace, and skilled staff measured the time they spent performing this task using the same stopwatch. Each participant was asked to perform the test twice, and the shortest time was recorded for the analysis.

5-time chair stand test

The chair stand test, also known as the chair rise test, was used as a proxy for leg muscle strength. Participants were asked to stand and sit in a chair five times as quickly as possible with their arms crossed over the chest, and skilled staff measured the time they spent performing this task using a stopwatch.

Short physical performance battery (SPPB)

The SPPB is a composite test that includes an assessment of gait speed and the balance and chair stand test [1]. For balance, participants were asked to remain standing with their feet as close together as possible, then move to a semi-tandem position (heel of one foot alongside the big toe of the other foot), and finally, to a tandem position (heel of one foot directly in front of the other foot). Each position was held for 10 s. Each test was scored from 0 (worst performance) to 4 (best performance), and a total score was obtained for the entire battery, which was the sum of all three tests and varied between 0 and 12 [2].

Untargeted metabolomics analysis of serum and stool samples

Serum samples were thawed on ice before extraction using 100 µL of serum and 300 µL of solvent (methanol/ACN (1:1)) with a 60-s vortex step before centrifugation at 4000 × *g*. After centrifugation, 150 µL of the reconstituted solution (methanol: H_2O = 1:1, v: v) was added to 300 µL of the supernatant with a 60-s vortex step before centrifugation at 4000 × *g* for reconstitution. For extraction, first, the feces samples were thawed on ice; next, 25 mg feces and 800 µL solvent (methanol/acetonitrile/ACN (2:2:1)) were ground together for 5 min before centrifugation at 25000 rpm for 15 min at 4°C. After centrifugation, 600 µL of the reconstituted solution (methanol: H_2O = 1:9, v: v) was added to 600 µL of the supernatant with a 60-s vortex step before centrifugation at 25000 rpm for reconstitution. The supernatants were collected for metabolomic profiling using LC-MS analysis. A quality control (QC) sample was prepared by mixing equal volumes (10 µL) of the supernatants of each sample to assess the analytical variability.

Untargeted metabolomics LC-MS analysis was performed on a Waters 2D UPLC (Waters, Milford, MA, USA) coupled to a Q-Exactive mass spectrometer (Thermo Fisher Scientific, Waltham, MA, USA) with a heated electrospray ionization (HESI) source and controlled using the Xcalibur 2.3 software program (Thermo Fisher Scientific, Waltham, MA, USA). Data were collected in both the positive- and negative-ion modes to improve metabolite coverage. In positive-ion mode, metabolites in the sample are converted to charged ions by electrospray ionization (ESI) or atmospheric pressure chemical ionization (APCI) plasma sources and then detected by mass spectrometry (MS). This mode is mainly used to detect metabolites with a strong polarity because these metabolites are prone to ion formation during electrospray ionization or atmospheric pressure chemical ionization. In negative-ion mode, electrospray ionization (ESI) or atmospheric pressure chemical ionization (APCI) plasma sources are also used, but the metabolites in the sample lose an electron to form negative ions before being detected by mass spectrometry. This mode is mainly used to detect metabolites with weak polarity or no polarity, because the negative ions formed by these metabolites after losing electrons are more stable. A Waters ACQUITY UPLC BEH C18 column (1.7 µm, 2.1 mm × 100 mm, Waters, USA) and a mobile phase consisting of 0.1% formic acid (A) and acetonitrile (B) in the positive mode and 10 mM ammonium formate (A) and acetonitrile (B) in the negative mode were used for chromatographic separation. The column was maintained at 45°C and the gradient conditions were as follows: 0–1 min, 2% B; 1–9 min, 2%–98% B; 9–12 min, 98% B; 12–12.1 min, 98 % B to 2 % B; and 12.1–15 min, 2% B. The flow rate was 0.35 mL/min and the injection volume was 5 µL. The mass spectrometric settings for the positive/negative ionization modes were as follows: spray voltage, 3.8/−3.2 kV; sheath gas flow rate, 40 arbitrary units; aux gas flow rate, 10 arbitrary units; aux gas heater temperature, 350°C; and capillary temperature, 320°C. The full scan range was 70–1050 m/z with a resolution of 70000, and the automatic gain control (AGC) target for MS acquisitions was set

to 3e6 with a maximum ion injection time of 100 ms. The top three precursors were selected for subsequent MS fragmentation with a maximum ion injection time of 50 ms and a resolution of 17500, the AGC was 1e5. The stepped normalized collision energies were set to 20 eV, 40 eV, and 60 eV.

16S rDNA microbiome analysis of stool samples

16S rDNA sequencing

DNA was extracted from all fecal samples using the MagPure Stool DNA KF kit B (Magen, Guangzhou, China) following the manufacturer's instructions. 16S rDNA was PCR-amplified and sequenced using the MiSeq system (Illumina, San Diego, CA, USA). The primer sequences used were F: 5′-GTGCCAGCMG CCGCGGTAA-3′ and R: 5′-GGACTACHVGGGTWT CTAAT-3′.

Operational taxonomic unit (OTU) clustering

The sequence reads with a similarity greater than 97% were identified and clustered into an OTU using the UPARSE software [3]. The representative OUT sequences were taxonomically classified using the Ribosomal Database Project (RDP) Classifier, and the community composition was analyzed for each taxonomic rank: domain, kingdom, phylum, class, order, family, genus, and species.

Rarefaction curve

A rarefaction curve was generated using the MOTHUR package (v1.31.2) [4] for the analysis of richness.

Diversity analysis

Alpha diversity analysis was performed to identify the complexity of the species diversity for each sample (group). To assess the diversity of samples (groups) for species complexity, beta diversity calculations were performed. Alpha and beta diversities were estimated using MOTHUR (v1.31.2) and QIIME (v1.8.0) [5] at the OTU level, respectively. The sample clustering was performed using QIIME (v1.8.0) based on UPGMA.

Statistical analysis

Statistical analysis was performed using the R (v3.4.1) software. Continuous variables are presented as medians (interquartile ranges), while categorical data are expressed as numbers and percentages (%). Significant differences in species and functions were evaluated using a Wilcox-test or Kruskal-Test. Linear discriminant analysis (LDA) coupled with effect size (LEfSe) was utilized to evaluate differentially abundant taxa.

Supplementary References

1. Guralnik JM, Simonsick EM, Ferrucci L, Glynn RJ, Berkman LF, Blazer DG, Scherr PA, Wallace RB. A short physical performance battery assessing lower extremity function: association with self-reported disability and prediction of mortality and nursing home admission. J Gerontol. 1994; 49:M85–94. https://doi.org/10.1093/geronj/49.2.m85 PMID:8126356

3. Edgar RC. UPARSE: highly accurate OTU sequences from microbial amplicon reads. Nat Methods. 2013; 10:996–8. https://doi.org/10.1038/nmeth.2604 PMID:23955772

4. Schloss PD, Westcott SL, Ryabin T, Hall JR, Hartmann M, Hollister EB, Lesniewski RA, Oakley BB, Parks DH, Robinson CJ, Sahl JW, Stres B, Thallinger GG, et al. Introducing mothur: open-source, platform-independent, community-supported software for describing and comparing microbial communities. Appl Environ Microbiol. 2009; 75:7537–41. https://doi.org/10.1128/AEM.01541-09 PMID:19801464

5. Caporaso JG, Kuczynski J, Stombaugh J, Bittinger K, Bushman FD, Costello EK, Fierer N, Peña AG, Goodrich JK, Gordon JI, Huttley GA, Kelley ST, Knights D, et al. QIIME allows analysis of high-throughput community sequencing data. Nat Methods. 2010; 7:335–6. https://doi.org/10.1038/nmeth.f.303 PMID:20383131
