# Supplementary material for: Serum metabolome and gut microbiome alterations are associated with low handgrip strength in older adults
Source: Aging (Albany NY). 2024 Feb 1;16(3):2638–56. doi: 10.18632/aging.205501 (PMC10911350; doi:10.18632/aging.205501)
Supplement: Supplementary Figure 1 [file aging-16-205501-s002.pdf]

SUPPLEMENTARY FIGURE

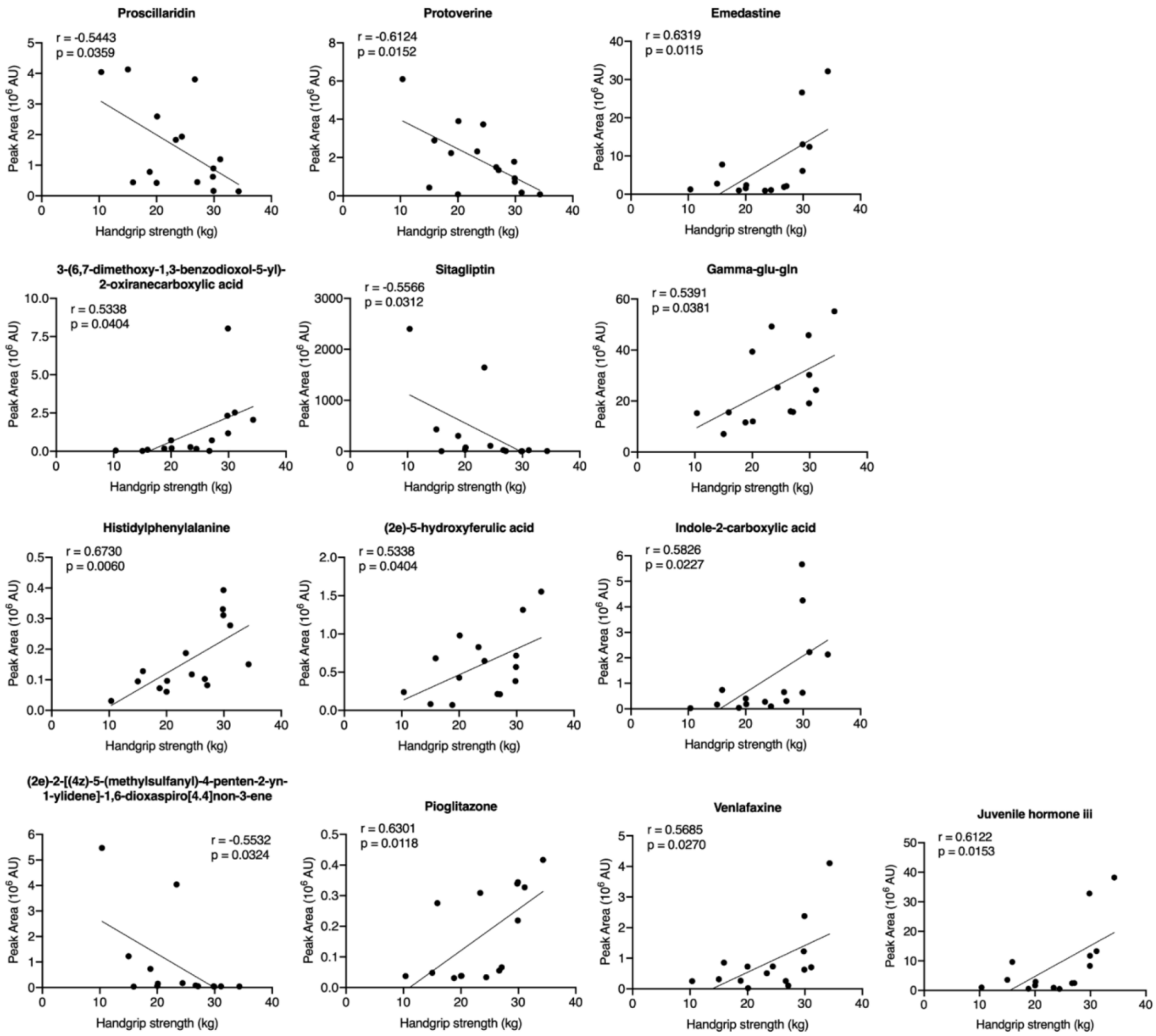

Supplementary Figure 1. Correlation analysis of the HGS value and differential metabolites in stool samples.
