# Supplementary material for: Serum metabolome and gut microbiome alterations are associated with low handgrip strength in older adults
Source: Aging (Albany NY). 2024 Feb 1;16(3):2638–56. doi: 10.18632/aging.205501 (PMC10911350; doi:10.18632/aging.205501)
Supplement: Supplementary Tables 2 and 4 [file aging-16-205501-s004.pdf]

SUPPLEMENTARY TABLES

Supplementary Table 2. Significantly enriched pathways of differential metabolites in serum samples between normal and low handgrip strength groups.

Metabolism pathway	Differential metabolites
Protein digestion and absorption	Indole; P-cresol; L-proline
Phenylalanine, tyrosine and tryptophan biosynthesis	Indole
Biosynthesis of unsaturated fatty acids	8z,11z,14z-eicosatrienoic acid; 11(z),14(z)-eicosadienoic acid; Linoleic acid
ABC transporters	D-ribose; D-(-)-mannitol; L-proline
Linoleic acid metabolism	8z,11z,14z-eicosatrienoic acid; Linoleic acid
Mineral absorption	L-proline
Pentose phosphate pathway	D-ribose
Central carbon metabolism in cancer	L-proline
Aminoacyl-tRNA biosynthesis	L-proline
Fructose and mannose metabolism	D-(-)-mannitol

Supplementary Table 4. Significantly enriched pathways of differential metabolites in fecal samples between normal and low handgrip strength groups.

Metabolism pathway	Differential metabolites
Cholesterol metabolism	Glycocholic acid; Glycochenodeoxycholate; Glycocholate
Primary bile acid biosynthesis	Glycocholic acid; Glycochenodeoxycholate
Purine metabolism	Hypoxanthine; Inosine
Bile secretion	Glycocholic acid; Glycochenodeoxycholate
Pantothenate and CoA biosynthesis	Panthenol
Tryptophan metabolism	L-3-hydroxykynurenine; 3-(indol-3-yl)-2-oxobutyric acid
One carbon pool by folate	Folinic acid
